# Developing an integrated depression and tuberculosis care pathway using a co-design approach in a low-resource setting

**DOI:** 10.1186/s13033-025-00670-0

**Published:** 2025-05-17

**Authors:** Olamide Todowede, Zara Nisar, Saima Afaq, Sushama Kanan, Aliya Ayub, Rumana Huque, Akhtar Hussain, Mudasser Shehzad, Najma Siddiqi

**Affiliations:** 1 Centre for Evidence Synthesis and Implementation Science, University of Birmingham, Birmingham, UK; 2 University of York, York, UK; 3 Khyber Medical University, Khyber Pakhtunkhwa, Pakistan; 4 Dept of Epidemiology and Biostatistics, School of Public Health, Imperial College, London, UK; 5 Ark Foundation, Dhaka, Bangladesh; 6 Provincial TB Control Program, Khyber Pakhtunkhwa, Pakistan; 7 Hull York Medical School, York, UK; 8 Bradford District Care NHS Foundation Trust, Bradford, UK

**Keywords:** LMICs, Depression, Tuberculosis, Codesign, Participatory approach, Pakistan, Bangladesh

## Abstract

**Background:**

Evidence suggests the use of a participatory approach for the improvement of TB care, however, there is limited evidence on how integrated depression screening and care could be delivered with TB services. Thus, this study co-designed an integrated care pathway for depression case finding and treatment in TB services, that can be delivered by non-mental health specialists within a low resourced settings.

**Methods:**

We conducted a total of 10 *‘co-design’* workshops with people with TB, carers, tuberculosis and mental health healthcare providers between June and August 2021 in Dhaka, Bangladesh and Peshawar, Pakistan. We adapted the ‘Hasso Plattner Institute of Design at Stanford University’ for our codesign process. Information gathered during the workshop, through recordings and contemporaneous notes taking, was collated, and analysed to develop the integrated care pathways and materials for impmenting the carepathway.

**Results:**

We co-designed and developed a culturally adaptable care pathway that integrates depression screening into tuberculosis (TB) care, working closely with people affected by TB and healthcare workers in primary, secondary, and tertiary care settings in Bangladesh and Pakistan. We used PHQ-9 only to identify and screen for depression among people with TB in Bangladesh, whereas both PHQ-2 and PHQ-9 were used for depression screening among the Pakistani population. A trained paramedic or laboratory technologist (Bangladesh) and DOTS Facilitator (Pakistan), working within the TB facilities were identified and agreed to deliver the integrated depression screening services.

**Conclusion:**

Stakeholders valued the opportunity to jointly design a care pathway. Iterative and coordinated working with these stakeholders enabled the researchers to understand better, explore and refine the co-design process. This approach assisted in mobilising knowledge about depression and integrating screening for depression within the existing usual TB care pathway, using the lived experience of people with TB and health workers’ expertise.

**Supplementary Information:**

The online version contains supplementary material available at 10.1186/s13033-025-00670-0.

## Introduction

Common mental health disorders such as depression and anxiety, are a significant cause of multimorbidity in people with TB in low and middle-income countries [1]. Comorbid depression and tuberculosis (TB) comorbidity have a bidirectional negative synergy, which is influenced by social, biological, and behavioural factors that collectively increase the risk for poor health and mental health outcomes [2, 3]. The mean weighted prevalence of depression is about 48.9% in people with TB [4], resulting in poorer health outcomes compared to people without TB, specifically in South Asia [5–9]. The prevalence of depression among people with TB is often associated with socio-determinants of health such as poverty, behavioural and biological factors [10, 11]. The management of this comorbidity requires a more patient-centred, holistic and multi-sectoral health systems approach is needed [10]. Economic evaluation studies indicate that every dollar invested in addressing depression and anxiety, generates a substantial global return on investment, emphasizing the compelling cost-effectiveness of integrating mental health and TB services [12, 13]. Yet there is an unmet need to develop high-quality evidence addressing TB-related mental health comorbidity, especially in low and middle-income countries [14].

In most LMIC settings, demand for mental health services is high, and there remains a persistent scarcity of financial resources, human capacity and inadequate infrastructure, posing significant challenges to the effective delivery and integration of mental health care [15, 16]. In these settings, where mental health service delivery remains inadequate, there is a critical need for culturally adaptable interventions and pragmatic strategies to address this gap. One effective approach involves integrating mental health care into existing health systems through task shifting, which empowers non-specialist healthcare workers to deliver mental health services. This strategy, combined with the development of culturally sensitive interventions, has the potential to significantly improve access to and the effectiveness of mental health care in low- and middle-income country (LMIC) settings. [16–20]. Similarly, integrating mental health services into primary health care, complemented by robust referral linkages, support, and supervision from secondary care, has been shown to enhance access to care and improve health outcomes. [21]. The WHO End TB Strategy 2015–2035 recommended the integration of mental health treatment into TB services as a key action to achieve the End TB target [22]. However, a review highlighted the limited adoption of person-centred approaches in the implementation of integrated interventions for common mental health disorders and TB services, particularly in LMIC settings [23].

In the development of culturally adaptable interventions, it is essential to incorporate the perspectives of diverse stakeholders during the initial stages of the design process. This approach enhances the acceptability, appropriateness, and comprehension of the intervention among service users, thereby fostering greater engagement and effectiveness [19]. A participatory approach such as ‘co-design’ allows both service users and other stakeholders to bring their experiences together in creating possible contextual solutions to improving the delivery and utilisation of health services [24, 25]. The involvement of service users at all levels of the mental health system using a participatory approach assists in generating practical knowledge about problems of concern and promotes personal and social change [26]. Despite the well-established benefits of involving service users in mental health systems in high-income countries [27], the engagement of service users and other stakeholders in shaping healthcare delivery is still emerging in low- and middle-income countries (LMICs) [28, 29]. Co-designing service delivery pathways has the potential to enhance the quality of care and increase patient satisfaction with health services. However, no study from South Asia has applied a co-design approach to develop an integrated care pathway for depression screening and management among tuberculosis patients. Therefore, we employed a co-design approach to develop a contextually appropriate strategy for depression case-finding and treatment within the tuberculosis (TB) care package. This intervention, referred to as ‘TB-D,’ includes tailored pathways and materials designed for implementation by non-mental health specialists in TB facilities in Bangladesh and Pakistan.

## Methods

This paper describes how a codesign team was developed and worked together to design an integrated depression-tuberculosis care pathway. It describes how potential service users and other stakeholders including healthcare workers and carers were involved as active partners in the development of the care pathway product.

### The project

This is the second phase of the communicable disease’s component of Improving outcomes in mental and physical multimorbidity and developing research capacity in the South Asia project (https://www.impactsouthasia.com/impact-group). The project aims to understand the feasibility of integrating depression screening and management services into tuberculosis services in South Asian countries. The first phase of the project explored the barriers and facilitators of integrating depression care into tuberculosis services in Bangladesh, India and Pakistan [30]. This provided the baseline information from which the codesign process was built with different stakeholders.

### Study setting, recruitment and participants

The study was undertaken at Khulna Mukti Seba Sangstha (KMSS) in Bangladesh, an implementing TB Control Program in Dhaka. The study took place at Khyber Medical University in Pakistan. All participants were aged 18 years and over. We recruited 42 stakeholders (23 Bangladesh and 19 Pakistan) to participate in the workshops through convenience and purposeful sampling. The recruitment rounds took place between June to August 2021. Mapping of the stakeholders was done to ensure representation of the full range of participants who were involved in the delivery or receipt of TB and mental health services. Patients, caregivers, public health experts, policymakers, and health care workers (mental health and TB experts) from primary secondary and tertiary care settings were identified and approached to participate in the co-design. In addition, we included stakeholders with expertise in public health and policy making and implementation. Interested participants confirmed their participation through word of mouth and response to memos sent to the health care workers including informed consent. Participants were remunerated for their time in each workshop attended. Participants could attend as many or as few workshops as desired. The participants included a diverse range of participants, encompassing individuals diagnosed with tuberculosis (TB), their caregivers, and healthcare professionals specializing in TB care. The healthcare professionals comprised TB clinic program managers, directly observed treatment (DOT) providers, psychologists, clinicians, medical officers with TB specialization, psychiatrists, pulmonologists, and non-governmental service providers. Additionally, policymakers, including officials from the National TB Program, were also engaged in the study.

### Study design approach

**Fig. 1:**
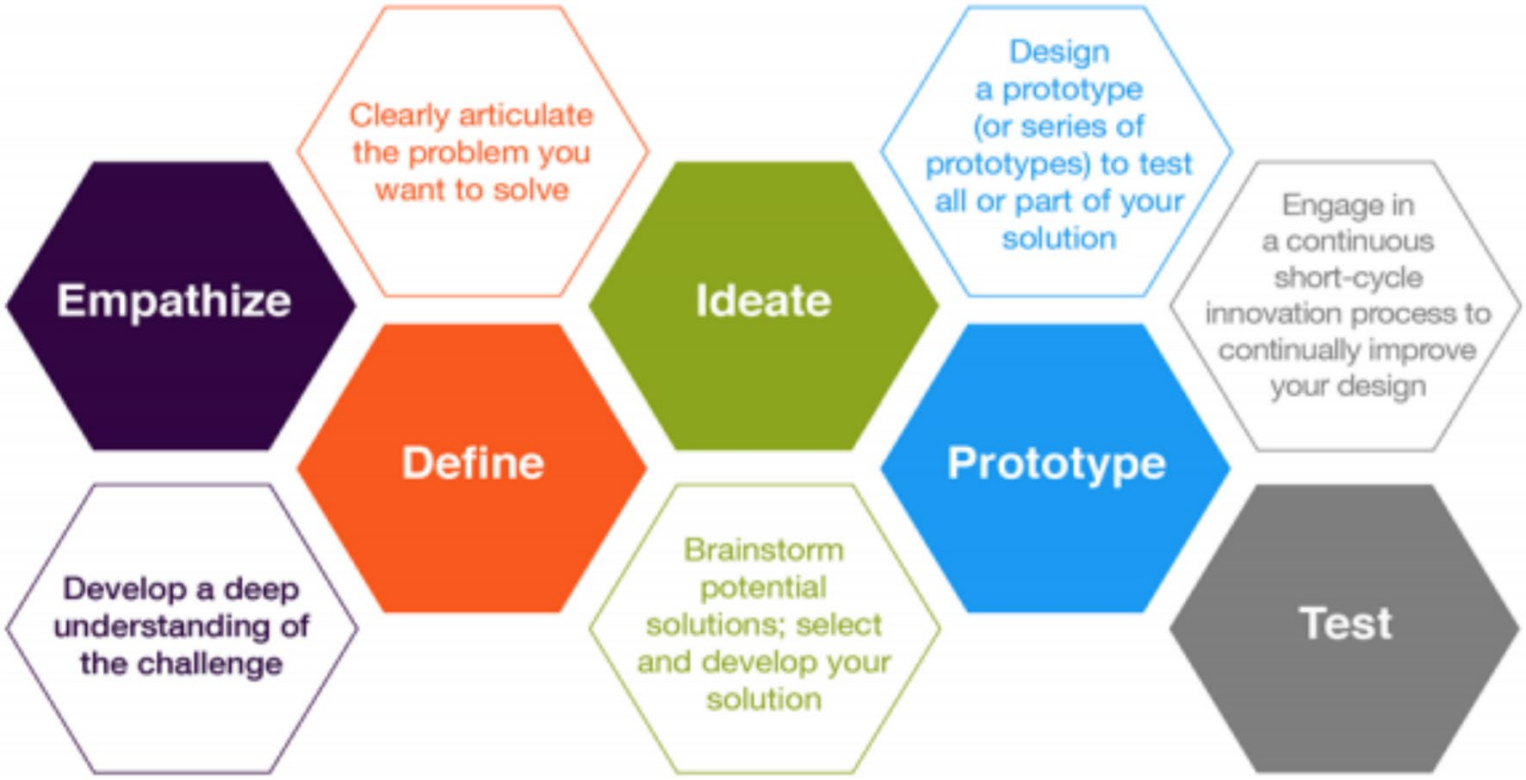
Hasso Plattner Institute of Design at Stanford University codesign process guide [31]

We adopted the theory-guided codesign process by the Hasso Plattner Institute of Design at Stanford University to design an integrated care pathway [31]. The co-design process includes a five-step approach (a) empathy, (b)define (c)ideate (d)prototype and (e) test—**(**Fig. 1**).** This co-design process utilises a participatory approach that is feasible, adaptable and flexible for the development of the care pathway product. The adoption of this co-design process was driven by its user-centred design thinking, iterative approach, and emphasis on multidisciplinary collaboration with a strong focus on end users. Additionally, it is both feasible and acceptable within culturally sensitive and local contexts [32]. The process goes beyond consulting with the stakeholders, we sought the active participation of service users with TB, healthcare workers and other stakeholders throughout the design process. The co-design process involves the equal partnership of individuals who work within the healthcare system as well as those who have lived the experience of a medical condition [24].

### Codesign workshops planning and attendance

The researchers (SK, MN) and (SA, ZN) conducted five co-design workshops in each country (Bangladesh and Pakistan) with people with TB, their carers, and clinical staff in TB clinics and mental health settings and TB programme funders (as policymakers). OT observed the virtual workshops in Bangladesh. They were guided by the codesign planning guide (Supplementary File 1: Co-design Supplementary material). OT designed workshop materials and trained researchers to conduct the workshops, OT and NS moderated the iterative process of analysing the workshop summary and development of the TB—depression care pathway prototype. Co-design workshops foster collaboration among stakeholders, creating a shared understanding of an issue while generating ideas informed by personal experience and evidence-based research [33].

The workshops in each country, brought together a diverse range of people with tuberculosis, their families/carers, and clinical staff in TB clinics and mental health settings and TB programme funders. A total of 23 participants in Bangladesh and 19 participants in Pakistan participated in the workshops. In Pakistan, the majority of participants attended at least four workshop sessions, indicating higher engagement compared to Bangladesh. In contrast, participation in Bangladesh was lower, with most attendees attending at most three of the five scheduled sessions (Table 1). Despite these differences, each session in both countries included representatives from various stakeholder groups, ensuring diverse perspectives were integrated into the care pathway development. The variation in workshop attendance was mainly due to COVID-19 pandemic restrictions, which affected participants' ability to join. To accommodate these challenges, the co-design workshops were conducted using a blended approach, combining in-person sessions and online participation via Zoom (https://www.zoom.com/). Each workshop lasted approximately 120 to 180 min and was audio-recorded for reference. Additionally, research team members took active notes during the sessions for reflection and documentation purposes. Researchers also reached out to participants who were unable to attend the workshops to update them on the outcomes and gather their perspectives. Additionally, all participants were invited to review and approve the final care pathway prototype.

**Table 1:** Attendance of participants by workshop session

Co-design workshop	Number of Participants Attendance	
	Bangladesh	Pakistan
Workshop 1	23	19
Workshop 2	15	19
Workshop 3	12	19
Workshop 4	8	19
Workshop 5	20	13

### Strategies for inclusive stakeholder engagement

We employed several strategies to encourage and enable active participation by all stakeholders: (i) training workshop facilitators who were fluent in the local language and empathetic toward stakeholders; (ii) ensuring no prior knowledge or specialized skills were required to participate; (iii) designing online workshops that did not require technical skills and could be accessed via mobile phones or from a convenient location; (iv) using ice-breaking activities to introduce stakeholders in a non-hierarchical manner and foster engagement; (v) conducting separate workshops for individuals with TB and their carers, distinct from sessions for healthcare professionals and policymakers; and (vi) starting workshops with introductions and icebreakers to build rapport and encourage critical thinking before engaging in research activities. These approaches helped create an equitable environment by amplifying all participants' voices, promoting shared decision-making, and removing barriers to participation.

The workshops always started with a quick introduction of all participants, and researchers stating the reasons and importance of the workshop sessions and a recap of the last workshop sessions.

### Stakeholder management

*Bangladesh specific*: Researchers took additional steps by following up privately with service users and carers after workshop sessions. This approach provided participants with a more comfortable space to share insights they may have hesitated to express in group discussions, ensuring their perspectives were fully integrated into the process.

*Pakistan-specific*: During the initial planning of the workshop sessions, the community panels expressed discomfort discussing issues in the presence of healthcare professionals. This was further compounded by male participants' reluctance to engage in discussions alongside female counterparts. However, this issue was addressed through additional discussions emphasizing the importance of gathering everyone's perspectives, regardless of gender, and the male participants agreed to this approach. To further address this power imbalance between the stakeholders, a separate workshop was held for patients and carers before the co-design process, ensuring they felt informed and confident to participate. During the workshop sessions, their contributions were prioritized to encourage active engagement. Additionally, discussions were conducted in the local language (Pashto) whenever possible to ensure clarity and inclusivity. To address these power dynamics, separate sessions were held for service users and carers, allowing them to share their perspectives independently. Feedback and contributions from healthcare professionals were then relayed to these groups, fostering a more inclusive and collaborative approach to developing the care pathway.

### Data analysis

We used an inductive, interactive data analysis approach to analyse the workshop activity content generated by stakeholders. Data Analysis was managed with Microsoft Word and open-source cross-platform graph drawing software (diagram.net) used to develop process flowcharts. Information gathered during these workshops, through recordings and notes taking, was synthesised, collated, and incorporated within the prototypes from both countries.

### Ethical considerations

The study was approved by the Health Science Research Governance Committee (*HSRGC/2020/418/D)* of the University of York. Ethical approval was received in all two countries through Ark Foundation in Bangladesh and KMU(Dir/KMU-EB/IPHSS/MM-001) in Pakistan. All participants in the co-design phase of the project gave written informed consent before taking part in the focus groups. All participant data was treated as confidential and stored securely at the University of York, United Kingdom server.

## Result

A total of 23 participants from Bangladesh and 19 participants from Pakistan (Table 2) attended the co-design workshops. The participants consisted of individuals affected by tuberculosis (TB), caregivers, healthcare workers (including TB clinic program managers, DOT providers, psychologists, clinicians, medical officers with TB specialization, psychiatrists, pulmonologists, and non-governmental service providers), and policymakers (such as officials from the National TB program). The individuals with TB had been undergoing treatment for 2 to 5 months, while the healthcare workers and policymakers had between 3 to 12 years of experience in providing TB and mental health services.

**Table 2:** Demographics of codesign participants (Bangladesh & Pakistan)

Participant	Male		Female		No. of workshops attended	
	Bangladesh (Total = 14)	Pakistan (Total = 12)	Bangladesh (Total = 9)	Pakistan (Total = 7)	Bangladesh	Pakistan
Carer	4	2	2	0	2	4
People with TB	4	2	5	1	2	4
Health Workers	1	4	0	4	3	5
Policy Makers	2	2	0	0	3	5
DOTS Facilitators	2	2	0	0	3	4
Psychologist/psychiatrist	1	0	0	1	2	5
Non-govt service provider	0	0	2	1	3	4

### Codesign Workshops

**Fig. 2:**
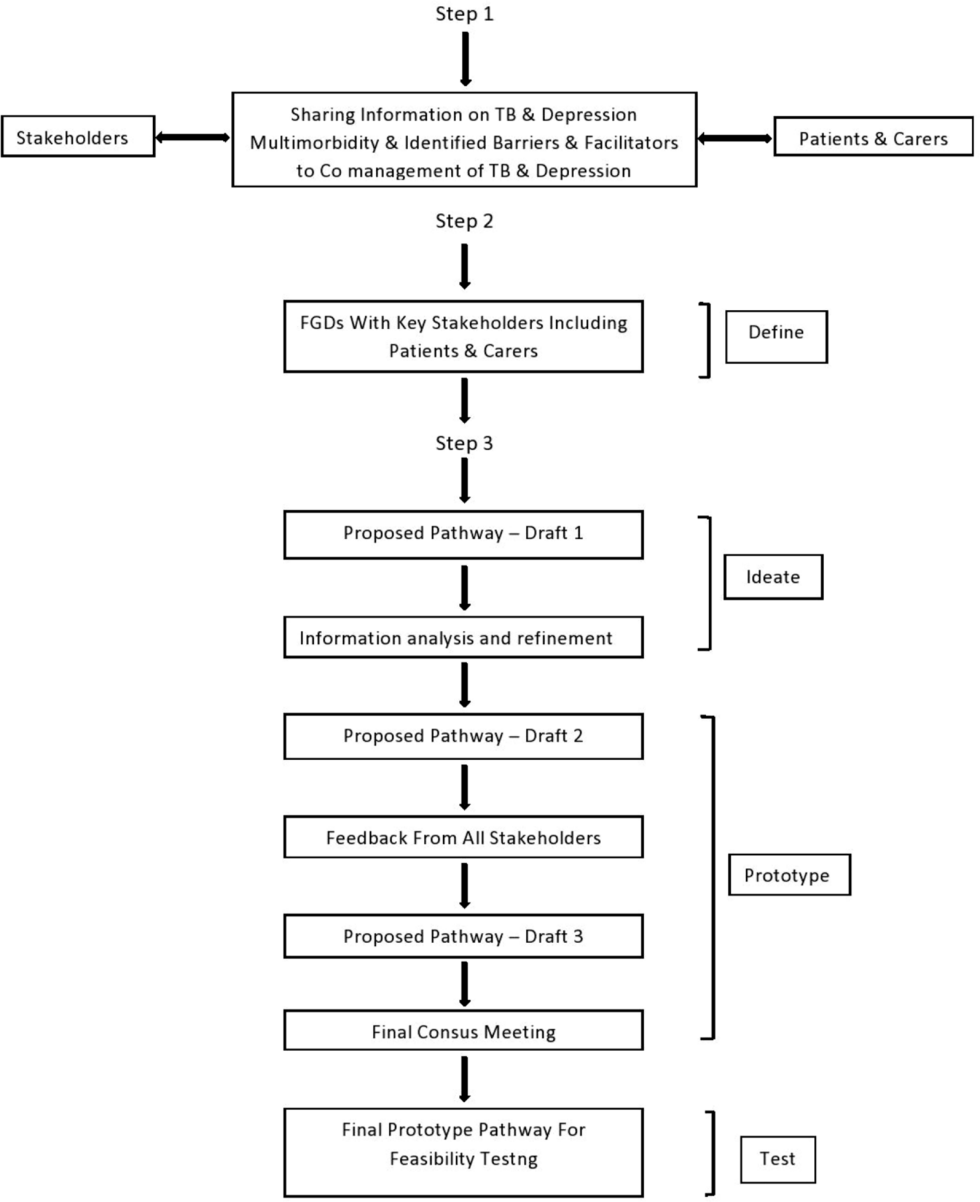
Co-designed process framework

The workshop content was similar across the two countries and the details of the workshop content are included below. Figure 2 illustrates the adopted codesign process for the development of the care pathway with all stakeholders and its related activities, the empathising stage (step 1) was done and has been reported in another publication[30].

### Workshop 1(Empathise)

Stakeholders were introduced to the importance and the need to co-design the care pathway and provided with an overview of the study's objectives in clear, accessible language. The contextual barriers and facilitators identified by stakeholders in the first phase of the study regarding the integration of depression care into tuberculosis services [30] were shared with workshop participants. Various communication methods were used to ensure clear understanding and meaningful engagement, allowing participants to interact in a way that suited their preferences. For health workers and policymakers, PowerPoint presentations were effective in the two countries. In Pakistan, a brief animated video was also used, while in Bangladesh, written materials and word of mouth were employed to communicate the study's objectives, as well as the identified barriers and facilitators, to individuals with lived experience of tuberculosis (patients) and their carers.

### Workshop 2 (Define)

Following workshop 1, stakeholders were allowed to share their lived experiences with tuberculosis (TB) and potential mental health conditions, offering feedback on the current TB care system and suggestions for integrating mental health services. A semi-structured questionnaire guided the discussion on (i) the barriers and facilitators to incorporating depression screening and treatment into TB care services, (ii) the development of a depression screening and treatment pathway within existing TB facilities, focusing on: (a) who will conduct the screenings, (b) when and how often patients will be screened (including TB health workers, people with TB, and their carers), (c) which tools will be used for depression screening (specifically by mental health professionals), and (d) how and where patients will be referred for mental health support if needed (involving both TB and mental health professionals). Additionally, healthcare workers mapped the nearest mental health facilities to the TB clinic, helping to assess the accessibility of these services for people with TB and their carers.

### Workshop 3 (Ideate)

**Fig. 3:**
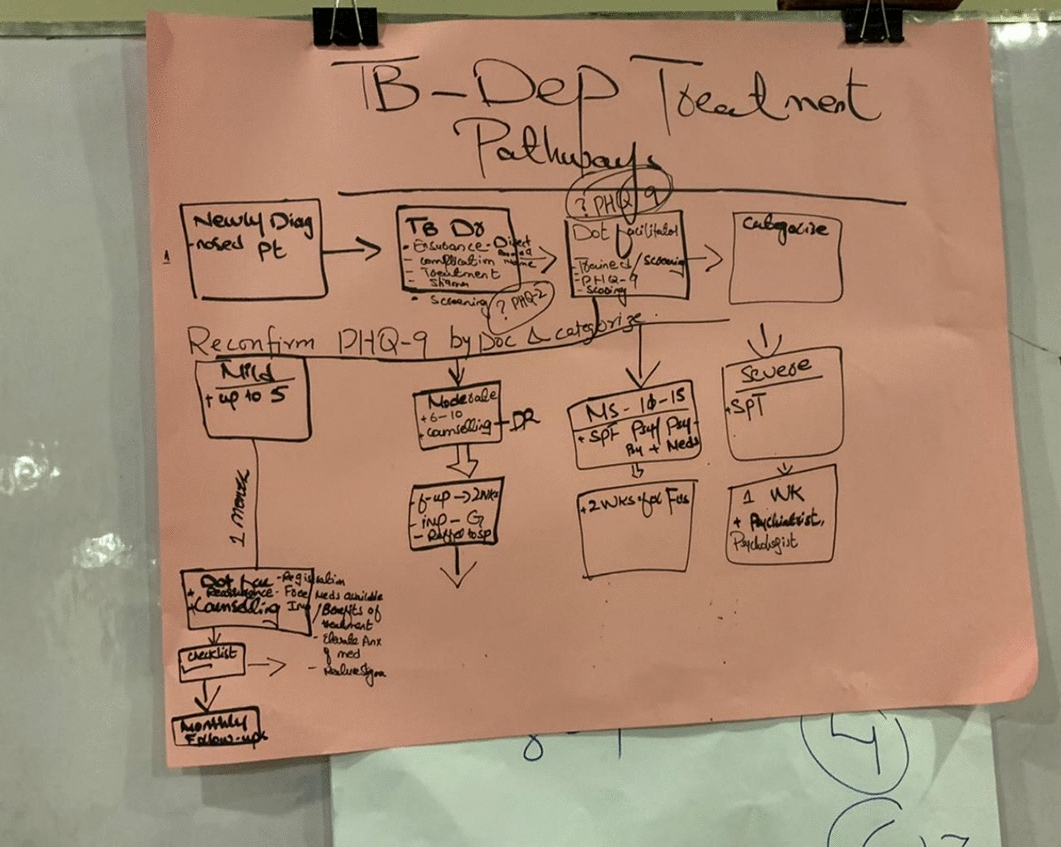
First draft of the Care-pathway designed by the workshop participants in Peshawar, Pakistan

**Fig. 4:**
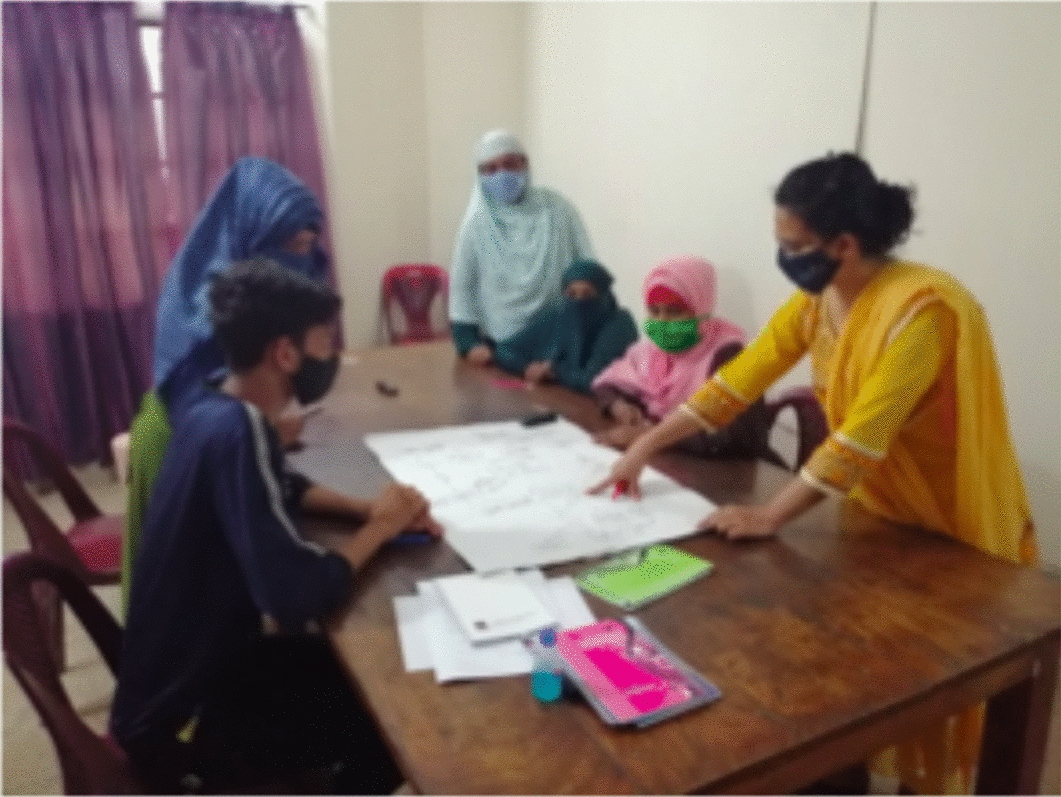
Consultation about the care-pathway design with participants in Dhaka, Bangladesh

In this workshop, participants built on their understanding from Workshop 1, along with their shared lived experiences and expertise from workshop 2, to generate ideas on how integrated depression and TB care could be achieved. All participants were encouraged to draw ideas of an integrated care pathway from their current care pathways, identifying potential opportunities and challenges, and suggesting ways to adjust the pathway accordingly. In Bangladesh, participants described their current care pathway and possible opportunities to allow integration of depression screening and management. This was conducted in the form of a structured discussion where researchers were actively developing the care pathway from participants' discussions and receiving iterative feedback from participants. Researchers ensured that participants' ideas were accurately captured and confirmed, reflecting what they intended to communicate, till a final and agreed version was developed. In Pakistan, participants were divided into two groups and tasked with mapping out a pathway (TB-D care pathway) for integrating depression screening and management into TB care services. Iterative refinements were made during the presentation and discussion sessions, leading to the final unified TB-D pathway produced and presented to the participants by the research team. The process allowed each group to present their prototype TB-D care pathway and further integrate their ideas to develop one final version **(**Figs. 3 and 4**)**. At this workshop, health workers identified the most suitable professionals for delivering depression screening. In Bangladesh, paramedics and lab technicians (they manage the DOT program) were recommended, while in Pakistan, the DOTS providers were deemed appropriate. As they are the first point of contact for people with TB, and the roles of the paramedics and lab technicians in Bangladesh and DOTS providers in Pakistan is to routinely screen for TB and conduct necessary tests, ensure medication delivery and adherence making them well-positioned to integrate depression screening into their role.

### Workshop 4 and 5 (prototype)

**Fig. 5:**
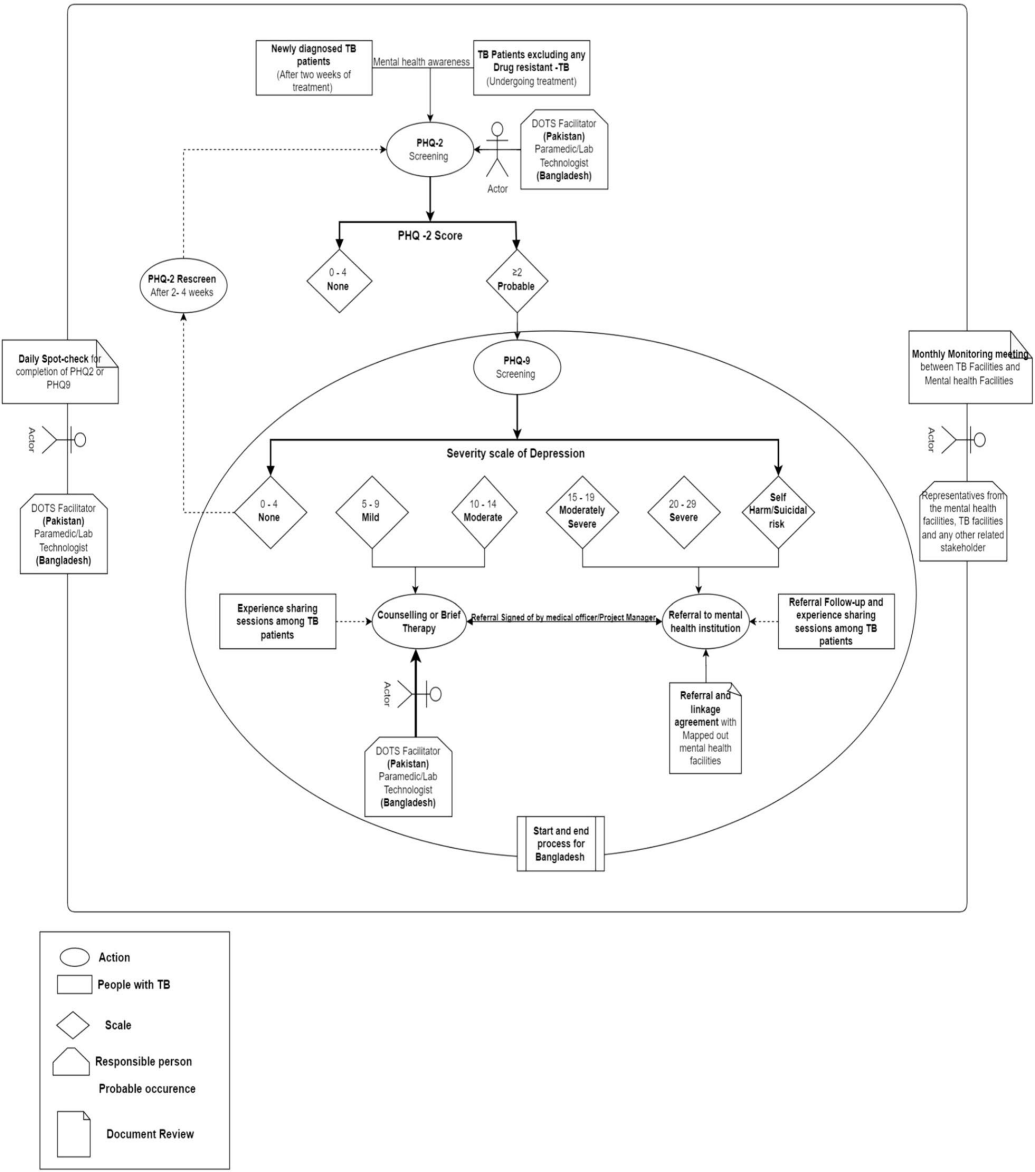
Integrated care pathway for depression case finding for tuberculosis patients

These workshop sessions were designed to present the developed initial prototypes generated throughout the past workshops and engagements between researchers and participants. Researchers gathered feedback on the needs and concerns of both end users (people with TB and their carers) and service providers to refine the initial prototypes. The sessions also focused on how communication and collaboration between TB clinics and mental health clinics could be effectively managed. A draft pathway for each country was presented to clinicians and mental health experts, who reviewed it for further refinement. Researchers then conducted additional checks of the care pathway with people with TB and their carers to assess accessibility and feasibility. Feedback was collected and incorporated into the care pathway for each country. As a research group, we aimed to develop a care pathway that is adaptable to different South Asia settings, therefore we identified similarities and differences between the pathways from each country. These findings were discussed among the researchers, leading to the integration of a single pathway for each country **(**Fig. 5**).**

### Feasibility testing

We trained health workers at the study sites using the depression module from the mhGAP-IG [34], teaching them how to screen for depression and use the developed care pathways. The training was led by a psychiatrist and the research team. The mhGAP-IG is comprehensive and suitable for use in low- and middle-income countries (LMICs) [35], and the trainers strictly followed the provided guide. To assess the effectiveness of the training and evaluate the health workers' ability to screen for depression, we conducted pre-and post-training assessments using the Adapted Revised Depression Attitude Questionnaire (R-DAQ) [36]. The results showed a significant improvement in health workers' knowledge and confidence in screening for depression among patients with tuberculosis (TB), with most participants strongly agreeing that their understanding had improved following the training. In the feasibility testing phase, we included monthly reflective meetings between representatives from the TB and mental health clinics, along with patient representatives. These meetings ensured the accurate and consistent implementation of the depression screening process and care pathway. They also provided a space for ongoing review, troubleshooting, and reinforcement of training principles to maintain fidelity in practice. The findings from the feasibility testing phase in Pakistan were reported in another paper, which highlighted the training's acceptability, practicality, and impact [37].

### Depression screening tool

The Patient Health Questionnaire (PHQ-2 and PHQ-9) were adopted for the screening for depression among our population. The PHQ -2 is used for inquiring about depression mood over the past two weeks, with a score ranging from 0 – 6 [38]. The PHQ–9 is used for screening, diagnosing and measuring the severity of depression mood [39]. The scores of PHQ-9 screening are specified into five categories of depression: no depression phase (0–4), mild [5–9], moderate [10–14], moderately severe [15–19] and severe [20–27].

### Delivery of the codesign care pathway (Prototype)

The integrated care pathway (Fig. 5) was designed for implementation over 3 months in two TB services at the primary level in Bangladesh and three TB services (one primary, one secondary, and one tertiary level) in Pakistan. During this period, all patients visiting the facilities will be screened for depression, regardless of their previous mental health history. Each study site was connected to a mental health unit for the referral of patients with severe mental health conditions that cannot be managed by non-mental health staff. These units will also provide support to the DOTS facilitators, paramedics, and laboratory technologists, should they require assistance. Both new and existing TB patients were included in the care pathway implementation. New TB patients were screened at their first visit after diagnosis, while existing TB patients were screened during subsequent visits after diagnosis or at medication pickup, with this recorded as their first depression screening. Screening at these two time points allows for monitoring of depression symptoms, considering that most patients attend the clinic quarterly. The stakeholders recommended that, in Pakistan, the DOTS facilitator, as the first point of contact, should carry out the initial screening using the PHQ-2, while in Bangladesh, the paramedic or laboratory technologist, who often serves as the first point of contact for TB patients, should administer the PHQ-9 screening. The prototype suggests that when individuals with TB are screened for depression, those who score ≤ 2 on the PHQ-2 will continue their usual TB care at their respective facilities. In Pakistan, those scoring > 2 will be further screened using the PHQ-9. However, due to limited human resources in Bangladesh, depression screening will begin directly with the PHQ-9.

Patients who score 0–4 on the PHQ-9 will be considered free of depression and will remain on their usual TB care pathway. Those with mild to moderate depression will receive counselling and reassurance from mhGAP-trained DOTS Facilitators, paramedics, and laboratory technologists. These staff members are trained to offer basic mental health support, and patients will be given a pamphlet outlining information on TB treatment, medication side effects, and available support services.

For patients with moderately severe or severe depression or those with suicidal thoughts or pregnancy, immediate action is required. These cases will be treated as emergencies and referred to a nearby mental health facility in the primary care setting, or to the mental health department of secondary or tertiary care facilities. A referral slip, signed by the attending doctor or facility manager, will be provided for patients requiring further mental health care. The mental health unit at the linked facility will be involved during the implementation phase to ensure appropriate referral pathways. We will ensure quality assurance, by conducting daily spot-checking by the doctors at the facility. Additionally, the research team will organise monthly reflective meetings with the mental health facilities, DOT Facilitators/Paramedics, Lab technologists, and doctors to share their experiences and discuss the challenges encountered during the feasibility testing phase.

## Discussion

### Main findings

The development of this integrated depression screening and management pathway followed a co-design process, primarily involving people with tuberculosis (TB), carers, healthcare workers, and policymakers. People with TB and their carers were particularly enthusiastic about having their opinions sought regarding their care, which was a new experience for them. Key considerations for sustainability included the feasibility of delivering depression screening by non-mental health specialists and fostering smooth collaborative working to ensure effective referral linkages to mental health facilities for those needing specialized care. These concerns were addressed through monthly meetings with TB clinics and mental health facilities to review and improve collaborative working practices where necessary. The co-design approach in developing this care pathway represents a significant advancement for integrated care systems, particularly in low- and middle-income countries (LMICs), which face resource limitations in improving population mental health services. The involvement of healthcare workers, people with TB, and their carers in the co-design process highlighted critical care gaps and underscored the need to integrate mental health services into physical health services. The development of the care pathway within the local context and its integration into the National TB program and healthcare service delivery in our study setting creates an opportunity for scaling up depression screening within the area. Service development and operational research are most effectively prioritized, designed, conducted, and replicated when integrated within the Ministry of Health and national programs [20, 40]. This approach ensures that care packages are tailored to local needs and remain feasible within the specific context. Also, the incorporation of a co-design approach into healthcare intervention development focuses on end users' perceptions. This care pathway is a promising tool for supporting both healthcare workers and individuals with TB in managing depression and suicidal ideation related to TB diagnosis or medication side effects [11, 41].

### Prior study

The co-design process demonstrates the potential to incorporate local populations' cultural and community needs into healthcare service delivery. However, further research is needed to explore factors that can assist in improving mental health services in these settings such as political will, better resource allocation, integrated care packages, and capacity building [15]. This includes training healthcare workers and ensuring consistent involvement and empowerment of patients, informal carers, and the wider community in decision-making. The use of a co-design process aligns with a realist approach to developing new tuberculosis practices, and emphasizes the necessity of participatory methods in creating complex interventions, as it facilitates connections among various stakeholders and bridges the gap between theory and practice [42]. The co-design approach enabled us to leverage the knowledge and expertise of workshop participants regarding depression screening within existing TB services. Our study employed a combination of virtual and in-person workshops for co-designing, an approach that allows flexible engagement with different stakeholders within LMIC settings [43]. Though our care pathway aligns with TB clinical practice guidelines and includes routine monitoring of depression symptoms, the prototype has only been tested on a small scale within our study project [37]. Further large-scale testing across diverse TB clinic settings is needed to evaluate its effectiveness, referral completion rates, cost-effectiveness, and the impact of task shifting—particularly when screening is conducted by paramedics and TB DOT facilitators.

Task shifting has been proposed as a cost-effective and efficient strategy to enhance integrated care and bridge the mental health gap in low-resource settings [44, 45]. Yet, task shifting should not be recognised as a universal solution to the health workforce shortage in LMICs [46]. Further research is needed to assess the scalability of the integrated care system and task shifting, including its sustainability, appropriateness, and potential challenges and opportunities [47]. This will help to better understand and adoption of task shifting within LMICs and how it can be effectively monitored and evaluated. Similarly, the cost and effects of integrated care indicate a reduction in health costs and improved health outcomes [48]. However, there is limited evidence regarding its impact on human resources, the specific population benefiting from the reduced healthcare costs, and the sustainability of integrated care in LMIC settings, especially considering that TB care is often aid-funded [49], while mental health services are frequently underfunded [50].

The feasibility of integrating routine depression screening into existing TB care pathways in our study settings presents potential implementation challenges. These challenges include limited awareness of depression, increased financial costs, a fragmented health system, limited resources, inadequate workforce training, logistical burden of referrals for individuals with TB and their caregivers, systemic barriers, resistance from health workers to task-shifting due to high workloads, and the absence of standardized mental health screening protocols and adequate training for healthcare workers [30, 37, 51]. A realist review suggests that these challenges can be addressed through a holistic approach that considers the interconnected elements within the health system and emphasizes their interdependence. This approach is particularly effective in low- and middle-income countries, where integrated diagnosis has been shown to improve health outcomes and patient care [52]. Strengthening health system governance, increased investment in digital health solutions for better coordination of multisectoral referrals and capacity-building initiatives to enhance health workers' competencies can assist in addressing implementation challenges [53]. Additionally, fostering community engagement, and leveraging task-shifting strategies can assist in addressing unmet mental health needs in low resource-constrained settings [54].

## Limitations

The co-design process in this study involved fewer patients and carers in the workshops compared to healthcare workers. The lower attendance of patients and their carers may have been due to the COVID-19 pandemic restrictions, during which the workshops were conducted. However, equal opportunities were provided for individuals with TB and health workers to share their experiences. While a larger number of participants would have allowed for a broader range of lived experiences to be captured and integrated into the co-design process, the insights gathered remain valuable. We ensured a balanced perspective from all stakeholders by actively seeking feedback from people with TB and their carers throughout the design process. Additionally, during feasibility testing, we ensured that patient perspectives were included as part of the care pathway review meeting conducted by healthcare workers. We recognize that some participants faced language barriers during the co-design process, as we used Bangla in Bangladesh and Urdu in Pakistan. To ensure inclusivity and address potential limitations, we employed various communication approaches to help participants understand the workshop content and feel confident sharing their views. For instance, in Pakistan, some participants preferred speaking Pashto, which our researchers understood and were able to translate, facilitating a more inclusive discussion. we encouraged individuals with TB to share their lived experiences by starting with individual tasks to maximize participation, followed by group discussions. Participants were also reassured that they could request retranslations if needed. The presence of bilingual researchers and psychologists during the workshop helped patients and their carers better understand the materials, ensuring more effective communication and engagement.

## Conclusion

Tuberculosis (TB) continues to have a high prevalence in low- and middle-income countries (LMICs), where the neglect of mental health issues among individuals with TB adversely affects both their quality of life and treatment outcomes. The interplay between TB and depression underscores the need for integrated care strategies that address comorbid physical and mental health issues. In settings with limited resources, such as those in LMICs, pragmatic solutions are required. The co-design process employed in this study facilitated the development of a culturally relevant and context-specific care pathway, which was acceptable and feasible for healthcare workers in TB care facilities in Bangladesh and Pakistan. At the time of this study, the integration of depression screening and treatment into TB care services was not implemented in the studied countries. However, through the co-design process, the need for such an integrated approach became evident across all stakeholder groups. The feedback garnered during the workshops was systematically communicated to provincial TB control programs to inform the potential implementation and feasibility testing of this integrated care pathway in subsequent phases of the study. As a project, we conducted a close out even across the two countries attended by diverse policy makers including clinicians, community panel members, National TB officials to advocate for integrated care system of TB and mental health services. The results of this study reinforce the critical importance of incorporating cultural adaptations early in the design of health interventions to improve their acceptability and effectiveness. By engaging both service providers and users, the study demonstrated that contextually tailored solutions can enhance the delivery and uptake of integrated mental health and TB care in resource-constrained settings. Future research should continue to evaluate the feasibility and impact of such integrated pathways in real-world settings to improve patient outcomes and optimize healthcare delivery.

## Supplementary Information

Additional file1 (DOCX 27 KB)

## Data Availability

The authors confirm that the data supporting the findings of this study are available within the article.

## Appendix 1

Example of the training manual for health workers in Bangladesh

### This training aims to equip you with the knowledge on how to recognize and treat depression in your usual setting. This is a three-day training.

Day 1 training manual

Screening for depression

- Screen every TB patient, either new or old, with PHQ9 whenever s/he will visit TB clinics for visiting doctor or taking DOTs
- Start the screening by filling up the proforma first. Copy the patient’s ID number from their TB card and write down it in the proforma. Complete the rest of the information.
- Then complete the PHQ9. Ask the patient all the 9 questions and make sure it is complete. Add the values and write down the total score and select the categories of depression according to total score.

**Table:**

Category	No depression	Mild depression	Moderate depression	Moderately severe depression	Severe depression
Score	0–4	5–9	10–14	15–19	20–27

## Management

- If the score is between 0–4, it means the person has no depression. This patient will be re-screened after 4 weeks when s/he will re-visit TB Clinic for taking DOTS medicine. The process will continue throughout the course of treatment if the scores remain same (0–4) at each time of rescreen.
- If the patient has mild/moderate depression, provide them psychological intervention according to WHO mhGAP depression module (you will be trained). Then schedule his/her next date for brief psychological intervention next week. Continue brief therapy for 6 consecutive weeks. There will be a total of 6 sessions of brief therapy. Then re-screen them with PHQ9.

## Referral procedure

- If a score indicates mild or moderate depression but presents suicidal tendency, self-harm behaviour or pregnancy, then instead of providing brief psychological intervention refer him/her to NIMH for treatment by a professional mental health specialist.
- If the patient has moderately severe/severe depression, refer him/her to National Institute of Mental Health(NIMH), using the PHQ9 referral slip.
- You will find a referral slip at the end of PHQ9. Write down the name of the patient and ID number(this is the same as the ID number on their clinic folder), fill up the slip and take a sign off from the project manager of TB clinic. Give that slip to the patient and make him/her understand the importance of referral and taking professional help from NIMH.
- The patient will take the referral slip to the NIMH and visit their outpatient department for further assessment or treatment as applicable.
- Write down the patient’s ID, phone number and date of referral in the referral register.
- Follow up the patient over phone within 1 week whether s/he takes the referral services.
- However, whether the patient takes referral services or not, rescreen him/her after 4 weeks when s/he visits TB clinic again for taking medicine.

## Other activities

Daily spot check of screening

- At the end of each day all the completed screening tools will be checked by the TB clinic project manager to see the completeness of the screening tool. She will go through all the completed screening tools and will look for any gap in the answer or miscalculation in total score. If she finds no discrepancy, she will sign off each screening tool.
- If there is any miscalculation or gap in the information, it will be asked to the person who completed it to check with the patient over phone and complete the proforma and PHQ9 screening tool.
- After full completion of the screening tool, it will be signed off by the project manager of TB clinic.

Fortnightly/Monthly clinical supervision

- One psychiatrist from NIMH will come for a supervision meeting every fortnight. All the personnels who are out the screening with PHQ 9 and providing brief psychological intervention will have to attend the meeting. The meeting will be fortnightly in the initial l two months, then it will be done monthly in the subsequent months. The supervision meeting sessions will be done remotely (online).
- The meeting is to assist in ensuring that the personnels are doing the right thing, also they will use that opportunity to discuss any issue or difficult situation with the NIMH representative. You can talk about any questions related to screening/brief therapy. You can share a case where you face difficulty and can take suggestions.

## Monthly group support session

Arrange a monthly group support session with patients who have taken brief therapy sessions from TB clinic or NIMH and those who have been referred to NIMH. The session will be held at TB clinic premise and will be conducted by paramedic and lab technologists. Some parts of the session will be led by patients as well. In this support session the patients who have taken services will share their experience with others. A mental health specialist from NIMH will also attend the session and will provide support to paramedic/lab technologists.

## Monthly courtyard meeting in the community

Arrange monthly courtyard meeting in the community where you will talk about the importance of mental health in TB patients and how to look for depression signs/ symptoms and where to seek help for such conditions.

## Data collection

- Keep all the patients’ document in the designated folder.
- Maintain a referral register.
- The following information will be collected from patients (the proforma will be shown in the training). The proforma will be filled up once during the 1st screening. In the follow up screening, only the follow up section of the proforma (consent, date and score) will be filled up in the same old proforma.
- Always maintain patients ID in proforma, PHQ9, referral slip and referral register as it is given in their TB card.

N.B. The depression care pathway diagram will be shown to the participants of the training to give them a visual representation of the procedure. The diagram will also be printed out and will be placed on their desk so that they can check whenever they want.

## Appendix 2

Guidelines of the care-pathway for integration of depression in the treatment of tuberculosis.

- New TB patient when comes for the first follow-up visit will be included in the care-pathway whereas the old TB patient with ongoing treatment will be included on the same day.
- The first point of contact of these patients will be the DOTS Facilitator.
- The DOTS Facilitator will screen them with the PHQ2 tool.
- If the score of PHQ2 is ≤ 2, the patient will return to the scheduled follow-up visit.
- On each follow-up visit, the patient will be re-screened with the PHQ2.
- If the score of PHQ2 is > 2, the Dots facilitator will screen the patient with PHQ9 tool.
- If the score of PHQ9 is 0–4, the patient will return to the scheduled follow-up and on each visit will be re-screened with the PHQ2 tool.
- If the score of PHQ9 is 5–9, the patient will be categorized as having Mild depression.
- If the score of PHQ9 is 10–14, the patient will be categorized as having moderate depression.
- If the score of the PHQ9 is 15–19, the patient will be categorized as having Moderately Severe depression.
- If the score of PHQ9 is 20–27, the patient is categorized as having Severe Depression.
- Daily Spot checking will be done by the Doctor at the facility.
- For Mild and Moderate Depression, Counselling will be done by the DOTS facilitator.
- In case of any red flag, like suicidal ideation or pregnancy, the Patient will be referred to a nearby Mental Health Facility in case of Primary Care Facility. For secondary Care facility and tertiary care facility the patient will be referred to the mental health department of the facility.
- During counselling the DOTS Facilitator will reassure the patient of:
- Free Medications will be available for treatment.
- Benefits of treatment.
- Reassurance that it is a treatable disease.
- Possible side effects.
- For Moderate and Severe Depression, Primary Health Facility will refer the patients to nearby Mental Health Facility. The referral slip will be signed by the Doctor at the center and then the patient will be referred.
- For Secondary and Tertiary Care Facility, the patients will be referred to the Mental Health Unit of their respective facilities. Collaborative management plans will be made for patients involving a psychologist.
- Referral Follow-up will be done by the Research Assistant of the team.
- Monthly Experience sharing will be done between the mental health facilities involved (psychiatrists, psychologists, trainee medical officers) Dots Facilitators, Doctors at the 3 sites and the research team.
- Research Team arranges review and coordination meeting with mental health and TB clinics.

## References

[1] Academy of Medical Sciences. Multimorbidity: A priority for global health research (2018). https://acmedsci.ac.uk/file-download/82222577 Accessed 15 Feb 2025

[2] Ambaw F, Mayston R, Hanlon C, Alem A. Depression among patients with tuberculosis: determinants, course and impact on pathways to care and treatment outcomes in a primary care setting in southern Ethiopia–a study protocol. BMJ Open. 2015;5(7): e007653. 26155818 10.1136/bmjopen-2015-007653 PMC4499723

[3] Sweetland A, Kritski A, Oquendo M, Sublette M, Norcini Pala A, Silva L, et al. Addressing the tuberculosis–depression syndemic to end the tuberculosis epidemic. Int J Tuberc Lung Dis. 2017;21(8):852–61. 28786792 10.5588/ijtld.16.0584 PMC5759333

[4] Sweetland A, Oquendo M, Wickramaratne P, Weissman M, Wainberg M. Depression: a silent driver of the global tuberculosis epidemic. World Psychiatry. 2014;13(3):325. 25273311 10.1002/wps.20134 PMC4219079

[5] Oh K, Choi H, Kim E, Kim H, Cho S. Depression and risk of tuberculosis: a nationwide population-based cohort study. Int J Tuberc Lung Dis. 2017;21(7):804–9. 28633706 10.5588/ijtld.17.0038

[6] Pachi A, Bratis D, Moussas G, Tselebis A. Psychiatric morbidity and other factors affecting treatment adherence in pulmonary tuberculosis patients. Tuberc Res Treat. 2013. 10.1155/2013/489865. 23691305 10.1155/2013/489865 PMC3649695

[7] Mason PH, Sweetland AC, Fox GJ, Halovic S, Nguyen TA, Marks GB. Tuberculosis and mental health in the Asia-Pacific. Australas Psychiatry. 2016;24(6):553–5. 27206468 10.1177/1039856216649770 PMC5332205

[8] Husain MO, Dearman SP, Chaudhry IB, Rizvi N, Waheed W. The relationship between anxiety, depression and illness perception in tberculosis patients in Pakistan. Clin Pract Epidemiol Ment Health. 2008;4:1–5. 18302758 10.1186/1745-0179-4-4 PMC2288599

[9] Balaji AL, Abhishekh HA, Kumar NC, Mehta RM. Depression in patients with pulmonary tuberculosis in a tertiary care general hospital. Asian J Psychiatr. 2013;6(3):251–2. 23642985 10.1016/j.ajp.2012.12.017

[10] Mendenhall E, Kohrt BA, Norris SA, Ndetei D, Prabhakaran D. Non-communicable disease syndemics: poverty, depression, and diabetes among low-income populations. The Lancet. 2017;389(10072):951–63. 10.1016/S0140-6736(17)30402-6 PMC5491333 28271846

[11] Issa BA, Yussuf AD, Kuranga SI. Depression comorbidity among patients with tuberculosis in a university teaching hospital outpatient clinic in Nigeria. Ment Health Fam Med. 2009;6(3):133. 22477903 PMC2838651

[12] Brunier A, Mayhew M. Investing in treatment for depression and anxiety leads to fourfold return. Geneva: World Health Organization; 2016.

[13] Chisholm D, Sweeny K, Sheehan P, Rasmussen B, Smit F, Cuijpers P, et al. Scaling-up treatment of depression and anxiety: a global return on investment analysis. Lancet Psychiatr. 2016;3(5):415–24. 10.1016/S2215-0366(16)30024-4 27083119

[14] Fonseka N, Khan Z, Lewis M, Kibria Z, Ahmad F, Khan MF, et al. Cognitive therapy for depression in tuberculosis treatment: protocol for multicentre pragmatic parallel arm randomised control trial with an internal pilot. BMJ Open. 2024;14(6): e083483. 38889941 10.1136/bmjopen-2023-083483 PMC11191785

[15] Rathod S, Pinninti N, Irfan M, Gorczynski P, Rathod P, Gega L, et al. Mental health service provision in low-and middle-income countries. Health Serv Insights. 2017;10:1178632917694350. 28469456 10.1177/1178632917694350 PMC5398308

[16] Sweetland AC, Oquendo MA, Sidat M, Santos PF, Vermund SH, Duarte CS, et al. Closing the mental health gap in low-income settings by building research capacity: perspectives from Mozambique. Ann Glob Health. 2014;80(2):126–33. 24976551 10.1016/j.aogh.2014.04.014 PMC4109687

[17] Ward K, Marimwe C, Parker MB, Dube LT. Towards integrated mental health services in low-income and middle-income countries: organisation of primary healthcare providers–a scoping review protocol. BMJ Open. 2024;14(2): e079854. 38382953 10.1136/bmjopen-2023-079854 PMC10882400

[18] Ndetei DM, Mutiso V, Osborn T. Moving away from the scarcity fallacy: three strategies to reduce the mental health treatment gap in LMICs. World Psychiatr. 2023;22(1):163. 10.1002/wps.21054 PMC9840495 36640407

[19] Ferrer-Wreder L, Sundell K, Mansoory S, editors. Tinkering with perfection: theory development in the intervention cultural adaptation field Child & Youth Care Forum. Cham: Springer; 2012.

[20] Walley J, Khan MA, Witter S, Haque R, Newell J, Wei X. Embedded health service development and research: why and how to do it (a ten-stage guide). Health Res Policy Syst. 2018;16:1–8. 30045731 10.1186/s12961-018-0344-7 PMC6060510

[21] Funk M, Saraceno B, Drew N, Faydi E. Integrating mental health into primary healthcare. Ment Health Fam Med. 2008;5(1):5–8. 22477840 PMC2777555

[22] Gonzalez J, Gastellanos L, Kamp N. Practical guide to improve quality TB patient care: a participatory approach. Pan American Health Organization: WHO; 2008.

[23] Janse Van Rensburg A, Dube A, Curran R, Ambaw F, Murdoch J, Bachmann M, et al. Comorbidities between tuberculosis and common mental disorders a scoping review of epidemiological patterns and person-centred care interventions from low-to-middle income and BRICS countries. Infect Dis Poverty. 2020;9:1–18. 31941551 10.1186/s40249-019-0619-4 PMC6964032

[24] Ward ME, De Brún A, Beirne D, Conway C, Cunningham U, English A, et al. Using co-design to develop a collective leadership intervention for healthcare teams to improve safety culture. Int J Environ Res Public Health. 2018;15(6):1182. 29874883 10.3390/ijerph15061182 PMC6025638

[25] Singh DR, Sah RK, Simkhada B, Darwin Z. Potentials and challenges of using co-design in health services research in low- and middle-income countries. Glob Health Res Policy. 2023;8(1):5. 36915174 10.1186/s41256-023-00290-6 PMC10009993

[26] Schneider B. Participatory action research, mental health service user research, and the hearing (our) voices projects. Int J Qual Methods. 2012;11(2):152–65.

[27] Bate P, Robert G. Experience-based design: from redesigning the system around the patient to co-designing services with the patient. Qual Saf Health Care. 2006;15(5):307–10. 17074863 10.1136/qshc.2005.016527 PMC2565809

[28] Abayneh S, Lempp H, Hanlon C. Participatory action research to pilot a model of mental health service user involvement in an Ethiopian rural primary healthcare setting: study protocol. Res Involv Engagem. 2020;6(1):2. 31934350 10.1186/s40900-019-0175-x PMC6951014

[29] Yadav UN, Lloyd J, Baral KP, Bhatta N, Mehta S, Harris MF. Using a co-design process to develop an integrated model of care for delivering self-management intervention to multi-morbid COPD people in rural Nepal. Health Res Policy Syst. 2021;19(1):1–12. 33568139 10.1186/s12961-020-00664-z PMC7874656

[30] Todowede O, Afaq S, Adhikary A, Kanan S, Shree V, Jennings HM, et al. Barriers and facilitators to integrating depression care in tuberculosis services in South Asia: a multi-country qualitative study. BMC Health Serv Res. 2023;23(1):818. 37525209 10.1186/s12913-023-09783-z PMC10391993

[31] An Introduction to Design Thinking PROCESS GUIDE [Internet]. Stanford d.school. 2017 https://web.stanford.edu/~mshanks/MichaelShanks/files/509554.pdf. Accessed 10 Apr 2023

[32] Yadav UN, Lloyd J, Baral KP, Bhatta N, Mehata S, Harris M. Evaluating the feasibility and acceptability of a co-design approach to developing an integrated model of care for people with multi-morbid COPD in rural Nepal: a qualitative study. BMJ Open. 2021;11(1): e045175. 33472791 10.1136/bmjopen-2020-045175 PMC7818838

[33] Vargas C, Whelan J, Brimblecombe J, Allendera S. Co-creation, co-design and co-production for public health: a perspective on definitions and distinctions. Pub Health Re Pract. 2022. 10.1706/phrp3222211. 10.17061/phrp3222211 35702744

[34] Organization WH. mhGAP training manuals for the mhGAP intervention guide for mental, neurological and substance use disorders in non-specialized health settings. Geneva: World Health Organization; 2017. 23741783

[35] Keynejad RC, Dua T, Barbui C, Thornicroft G. WHO mental health gap action programme (mhGAP) intervention guide: a systematic review of evidence from low and middle-income countries. BMJ Ment Health. 2018;21(1):30–4. 10.1136/eb-2017-102750 PMC10283403 28903977

[36] Haddad M, Menchetti M, McKeown E, Tylee A, Mann A. The development and psychometric properties of a measure of clinicians’ attitudes to depression: the revised depression attitude questionnaire (R-DAQ). BMC Psychiatry. 2015;15:1–12. 25653089 10.1186/s12888-014-0381-x PMC4321322

[37] Afaq S, Ayub A, Faisal MR, Nisar Z, Zala, Rehman A, et al. Depression care integration in tuberculosis services: a feasibility assessment in Pakistan. Health Expect. 2024;27(1): e13985. 39102704 10.1111/hex.13985 PMC10849063

[38] Kroenke K, Spitzer RL, Williams JB. The patient health questionnaire-2: validity of a two-item depression screener. Med Care. 2003;41(11):1284–92. 14583691 10.1097/01.MLR.0000093487.78664.3C

[39] Kroenke K, Spitzer RL, Williams JB. The PHQ-9: validity of a brief depression severity measure. J Gen Intern Med. 2001;16(9):606–13. 11556941 10.1046/j.1525-1497.2001.016009606.x PMC1495268

[40] UNICEF, Organization WH. Framework for operations and implementation research in health and disease control programs. 2008.

[41] Ruiz-Grosso P, Cachay R, De La Flor A, Schwalb A, Ugarte-Gil C. Association between tuberculosis and depression on negative outcomes of tuberculosis treatment: a systematic review and meta-analysis. PLoS ONE. 2020;15(1): e0227472. 31923280 10.1371/journal.pone.0227472 PMC6953784

[42] Marchal B, Abejirinde I-OO, Sulaberidze L, Chikovani I, Uchaneishvili M, Shengelia N, et al. How do participatory methods shape policy? Applying a realist approach to the formulation of a new tuberculosis policy in Georgia. BMJ Open. 2021;11(6): e047948. 34187826 10.1136/bmjopen-2020-047948 PMC8245474

[43] Knowles S, Hays R, Senra H, Bower P, Locock L, Protheroe J, et al. Empowering people to help speak up about safety in primary care: using codesign to involve patients and professionals in developing new interventions for patients with multimorbidity. Health Expect. 2018;21(2):539–48. 29266797 10.1111/hex.12648 PMC5867321

[44] Semrau M, Lempp H, Keynejad R, Evans-Lacko S, Mugisha J, Raja S, et al. Service user and caregiver involvement in mental health system strengthening in low-and middle-income countries: systematic review. BMC Health Serv Res. 2016;16(1):1–18. 26931580 10.1186/s12913-016-1323-8 PMC4774091

[45] Last BS, Buttenheim AM, Futterer AC, Livesey C, Jaeger J, Stewart RE, et al. A pilot study of participatory and rapid implementation approaches to increase depression screening in primary care. BMC Fam Pract. 2021;22(1):1–26. 34784899 10.1186/s12875-021-01550-5 PMC8593851

[46] Peresu E, Heunis JC, Kigozi NG, De Graeve D. Task-shifting directly observed treatment and multidrug-resistant tuberculosis injection administration to lay health workers: stakeholder perceptions in rural Eswatini. Hum Resour Health. 2020;18:1–12. 33272307 10.1186/s12960-020-00541-4 PMC7712623

[47] Yankam BM, Adeagbo O, Amu H, Dowou RK, Nyamen BGM, Ubechu SC, et al. Task shifting and task sharing in the health sector in sub-Saharan Africa: evidence, success indicators, challenges, and opportunities. Pan Afr Med J. 2023. 10.1160/pamj.2023.46.11.40984. 10.11604/pamj.2023.46.11.40984 PMC10683172 38035152

[48] Rocks S, Berntson D, Gil-Salmerón A, Kadu M, Ehrenberg N, Stein V, et al. Cost and effects of integrated care: a systematic literature review and meta-analysis. Eur J Health Econ. 2020;21:1211–21. 32632820 10.1007/s10198-020-01217-5 PMC7561551

[49] Organization WH. Financing for TB prevention, diagnostic and treatment services. Geneva: WHO; 2021.

[50] MacArthur G. Challenges and priorities for global mental health research in low-and middle-income countries. Symposium report. 2008.

[51] Organization WH. Continuity and coordination of care: a practice brief to support implementation of the WHO Framework on integrated people-centred health services. 2018.

[52] Gwaza G, Plüddemann A, McCall M, Heneghan C. Integrated diagnosis in Africa’s low-and middle-income countries: what is it, what works, and for whom? A realist synthesis. Int J Integr Care. 2024;24(3):20. 39280804 10.5334/ijic.7788 PMC11396343

[53] Druetz T. Integrated primary health care in low-and middle-income countries: a double challenge. BMC Med Ethics. 2018;19:89–96. 29945623 10.1186/s12910-018-0288-z PMC6020002

[54] Hoeft TJ, Fortney JC, Patel V, Unützer J. Task-sharing approaches to improve mental health care in rural and other low-resource settings: a systematic review. J Rural Health. 2018;34(1):48–62. 28084667 10.1111/jrh.12229 PMC5509535

