# Supplementary material for: Developing an integrated depression and tuberculosis care pathway using a co-design approach in a low-resource setting
Source: Int J Ment Health Syst. 2025 May 17;19:15. doi: 10.1186/s13033-025-00670-0 (PMC12084970; doi:10.1186/s13033-025-00670-0)
Supplement: Supplementary file 1 — Additional file1 (DOCX 27 KB) [file 13033_2025_670_MOESM1_ESM.docx]

**WS2: CODESIGN WORKSHOP PLANNING GUIDE**

**The purpose of the codesign workshop:**

1. The purpose of the workshop is for stakeholders to co-design care pathways and materials to support case detection and treatment of depression in tuberculosis patients attending study sites.

The following processes will be followed in planning and conducting the workshop session(s).

1. **Stakeholder Mapping:** This process will involve listing all the stakeholders (considering the primary stakeholders, key actors and intermediate actors) that are relevant to achieving the purpose of the co-design workshop. Their ability, relevance, and impact to contribute to the purpose of the workshop and availability to attend the workshop will be assessed. The inclusion criteria for the selection of stakeholders will be people that are working on tuberculosis care and management, as well as people affected and infected by TB - depression within the selected study sites (***Please see table 1***).

**Responsibility:** The country site and University of York team.

***Table 1: List of Stakeholder portfolio***

| **Primary Stakeholders** | **Key Actors** | | **Intermediate Actors** |
| --- | --- | --- | --- |
| TB Patients, community panels, support groups, TB activist groups, TB patients carers | Senior health practitioners | Junior Health Practitioner s | Not for Profit Organisations and others |
| *List* | *List* | *List* | *List* |
| *List* | *List* | *List* | *List* |

1. **Snowballing:** The contacted stakeholders will be asked to suggest and assist in mapping out more stakeholders (if we have missed out any), the suggested stakeholders will be contacted for their consent to participate in the study. Also suggested stakeholders that had been mentioned during the WS2 workshop will be contacted. Some of the participants of WS1 will be contacted, based on their expected impact on the codesigning.
2. **Final Stakeholder mapping:** The final list of stakeholders will be drawn and finalized. The stakeholders will be contacted and informed about the study.
   1. **4. Contacting and consent:** The stakeholders will be contacted by phone or in-person and informed about the study and we will seek their consent to participate in the study. ***Online workshops:*** The information sheet and consent form (For online workshops) will be shared with the stakeholders. Stakeholders that indicated an interest in the study will be sent the consent form, stakeholders that consent and return the signed forms will be contacted about the date and form of the workshop. *Facilitators should follow -up with calls and further discussions if required by the proposed participant.*
   2. ***Physical workshops:*** The proposed stakeholders will be contacted through email, memos, phone calls or word of mouth with the information details, workshop date and venue. The workshop participants will sign off the consent form before the start of the workshop.

*The facilitator must ensure that all the participants read and understand the information sheet, if yes, please confirm that all consenting participants sign the consent form.*

**WS2: CODESIGN WORKSHOP FACILITATION FOR ALL STAKEHOLDERS**

**Name of Facilitator:**

**Date and Time of Facilitation:**

**The venue of Workshop:**

**Format of the workshops: Online (name the mode) or Physical contact.**

**Introduction**

"Good morning. I am *(facilitator introduces him/herself),* and I will be facilitating this codesigning workshop.

This workshop is being conducted to codesign care pathways and materials to support case detection and treatment of depression in tuberculosis patients attending study sites. This workshop will inform our research that aimed at identifying and managing depression in tuberculosis.

As stated in the information sheet, I will be recording our conversation. The purpose of this is so that I can get all the details, and at the same time be able to carry on an attentive conversation with you. I assure you that all your comments will remain confidential and anonymised at the point of translating our conversation.

*The facilitator confirms that all participants have read, understood the information sheet, consented and signed the consent form. If yes, the stakeholder workshop can commence.*

**Workshop 1:** Stakeholder sensitisation and a co-interpreting session: In this section, the study’s objectives and expected outcomes will be discussed to develop a shared understanding amongst researchers and stakeholders of the co-design approach, and to share findings from workstream 1.

**Workshop 2-4:** These sections will be used in designing the pathways and materials. All Stakeholders will be engaged to give ideas and feedback based on their lived experiences and practice. Iterative refinements will be made and presented to the participants until the final contents are agreed. As part of the process, participants will be asked to try out various materials as they are developed and provide feedback on their acceptability and feasibility.

**Workshop 5:** Prototyping: The drafts pathways and materials developed will be reviewed by the research team, and by clinicians and managers with expertise in mental health and tuberculosis and further refined in the light of their feedback.

(*Please ensure that participants are comfortable and that they can express themselves, stakeholders can be split into groups to brainstorm and share their thoughts, they can be given materials to note their discussions, recommendations and strategies, participants can be given activities to map out facilities and resources, thoughts and cultural context activities and plans. Let the participants know that there is no idea that is not worth discussing, so feel free to talk as much as you can within our limited time.*)
